# Prevalence of psychological and metabolic concerns in children with white coat and ambulatory hypertension

**DOI:** 10.3389/fneph.2026.1699959

**Published:** 2026-06-08

**Authors:** Anne E. Dawson, Vanessa Thiel, Andrew H. Tran, Camille S. Wilson, Mahmoud Kallash

**Affiliations:** 1 Department of Pediatric Psychology and Neuropsychology, Nationwide Children’s, Columbus, OH, United States; 2 Department of Pediatrics, The Ohio State University, Columbus, OH, United States; 3 Department of Nephrology, Northwestern University, Lurie Children’s Hospital, Chicago, IL, United States; 4 The Heart Center, Department of Pediatrics, The Ohio State University, Nationwide Children’s Hospital, Columbus, OH, United States; 5 Department of Nephrology and Hypertension, The Ohio State University, Nationwide Children’s, Columbus, OH, United States

**Keywords:** anxiety, children, hypertension, mental health, white coat hypertension

## Abstract

**Background:**

White Coat Hypertension (WCH), or elevated blood pressure in response to stress, is common in childhood. While studies showed that hypertension (HTN) can be associated with psychological and neurocognitive impairments, the prevalence of such concerns in children with WCH is not well studied. This study aimed to evaluate prevalence of metabolic and positive mental health screening in patients with WCH and hypothesized that those concerns are higher in patients with WCH compared to HTN, while they have less frequency of metabolic risk factors.

**Methods:**

This is a single-center cross- sectional study of patients referred to the pediatric nephrology outpatient clinics to assess hypertension. All patients completed a 24-hour ambulatory blood pressure monitor and classified as HTN vs WCH. Various patient and parent-reported psychosocial screening surveys were self-administered. Results of kidney function, lipid panel, and echocardiogram were collected (if available). Descriptive analyses were used to assess psychological and metabolic factors across patients, with specific focus on assessing the differences between patients with HTN versus WCH.

**Results:**

The study enrolled 552 patients (56.3% had HTN; 43.7% had WCH), and of the included patients: 472 completed the screening surveys (54.2% with HTN; 43.8% with WCH). There were no significant differences between groups across metabolic risk factors of primary HTN. Furthermore, there were no significant differences in prevalence of psychological markers between the groups; however, the general prevalence across all patients with screening positive for at least one mental health concern was high at 50.1%.

**Conclusions:**

Metabolic and mental health concerns, including inattention, anxiety, and depression are common in patients with WCH at a similar rate to HTN. This highlights the importance of considering psychosocial functioning testing when treating pediatric patients with hypertension disorders.

## Introduction

Hypertension (HTN) is a growing healthcare concern in children and adolescents. In the US, around 2 - 5% of children have HTN with increasing prevalence, especially in children with obesity and decreased level of physical activity (1, 2). HTN can result in significant adverse personal, economic, and social impacts, including missing work and school (1).

Consensus statements have increasingly recognized that causes of pediatric hypertension are multifaceted, including fetal exposures, physical fitness, weight gain, sleep, and environmental factors (3). At present, metabolic factors (such as obesity, dyslipidemia and metabolic syndrome) are the most considered factors to correlate with HTN, given that most cases of pediatric HTN are primary in nature (1, 2). Poorly controlled HTN is known to cause systemic complications including cardiovascular disease, retinopathy, and chronic kidney disease. On the other hand, and while it is less studied, children with HTN can also demonstrate neurocognitive vulnerabilities (4). Several comprehensive reviews highlight the pathophysiological mechanisms, and potential cognitive deficits present in pediatric patients with HTN (4–6). Indeed, children with primary hypertension have significantly lower performance on neurocognitive testing and increased concerns about attention compared to normotensive controls (4–9).

White coat hypertension (WCH) is also very common in children, with studies estimating that WCH accounts for 40-60% of elevated blood pressure (BP) at clinic visits (10, 11). WCH is diagnosed when BP measurements are elevated in the office but are normal at home and is best diagnosed with a 24-hour ambulatory blood pressure monitor (ABPM) (12). While HTN often needs to be treated with BP medications, guidelines do not currently support treating WCH with BP agents (12). WCH may be indicative of elevation in anxiety induced by the office blood pressure checks (13), and some studies suggest that children with WCH may have significantly more stress (14), perhaps expressing elevated BP through mood or conditioned anxiety responses. Despite that, WCH is not a benign phenomenon. Studies showed that children with WCH are more likely to eventually develop HTN (11, 15–17). Patients with WCH are also more likely to have left ventricular hypertrophy (LVH) compared to healthy controls (15, 18). However, the factors contributing to WCH in children are not well studied.

Pediatric hypertension disorders are complex and can have heterogenous presentations and thus, it may be difficult to parse apart psychological differences between the populations. Considering the different causes and treatment approach of pediatric patients with HTN versus WCH, it is reasonable to speculate that these patients may present differently. Thus, the current study aimed to characterize and compare the prevalence of psychosocial concerns and metabolic factors between patients with WCH and HTN, diagnosed per ABPM results in an outpatient pediatric nephrology clinic. We hypothesized that the prevalence of the psychosocial concerns would be higher in patients with WCH compared to those with HTN and that children with WCH would have fewer metabolic risk factors than the HTN group. The findings of this study could help inform recommendations for behavioral health screening and interventions in the management of pediatric hypertension.

## Methods

This is a single-center cross-sectional study. All children referred by their primary care providers and presented to the outpatient pediatric nephrology clinic for hypertension evaluation between 6/1/2020 to 8/31/2021 were enrolled. The pediatric nephrology clinic typically sees patients with hypertension concerns from early childhood through young adulthood, ages 4 to 21 years old. In some cases, the clinic will see older patients if they are more complex in nature or being followed by other clinics within the children’s hospital (e.g., for ongoing cerebral palsy care). At the initial visit, the patients received a 24-hour ambulatory blood pressure monitor (ABPM), administered by nephrology outpatient nurses. Patients and their parents were also evaluated for various psychosocial concerns and risks using standardized psychological screening surveys, which were handed to them at their ABPM visit check-in (see Measures section below). This study was approved by the hospital’s Institutional Review Board at Nationwide Children’s Hospital.

After patients were enrolled, we reviewed their electronic medical chart and extracted relevant clinical data (age, gender, self-reported race, self-reported social determinants of health, weight, height and body mass index [BMI]). For this study, a low BMI is considered at or below the 5^th^ percentile for age and sex, and a high BMI is considered at or above the 95^th^ percentile for age and sex (based on the percentile data provided by the Centers for Disease Control for BMI) (19). Available laboratory values were recorded if completed within 6 months around the date of the ABPM and included kidney function (specifically: serum creatinine to calculate the estimated glomerular filtration rate, blood urea nitrogen [BUN] and albumin), hemoglobin A1c (HbA1c), triglycerides, and low density lipoprotein cholesterol (LDL-C). If multiple lab values existed, the lab value that was closest in date to when the patient completed the ABPM was used. Kidney function was estimated based on the CKiD formula (20), as some of the patients did have CKD. A low eGFR was considered below 90 ml/min/1.73 m^2^, and a high eGFR (hyperfiltration) was considered above 120 ml/min/1.73 m^2^, for the purpose of this study.

For the ABPM study, we used SpaceLab machines that were programmed for a BP check every 20 minutes during the day, and every 30 minutes over night. A minimum of 40 BP readings over a 24 hour monitoring period was required to consider the ABPM results as adequate. For the purposes of this project, we included systolic and diastolic BP load and percentage of nocturnal BP dipping as well as the mean arterial pressure (MAP). Additionally, the diagnosis of the patient (ambulatory HTN or WCH) was based on the ABPM results recorded and the nephrologists’ clinical judgment. HTN was diagnosed when either daytime, nighttime and/or average BP were ≥95^th^ percentile for sex and height for children <13 years old. For youth ≥13 years old, HTN was diagnosed if the daytime average was ≥ 130/80 mmHg, nighttime average of ≥110/65, or a 24-hour average of ≥125/75 mmHg, consistent with the American Heart Association 2022 scientific statement (12). WCH was defined by having elevated manual BP in the office (> 95% for age, height, sex for children <13 years old or above 130/80 mmHg in children ≥13 years old), while results of ABPM were normal (12). Systolic and or diastolic load is the percentage of systolic/diastolic blood pressure readings that exceed the hypertension threshold (> 95% for children < 13 years of age, >130/80 mmHg daytime, >110/65mmHg nighttime for patients > 13).

Results of their echocardiogram were also included if completed within the last year of the ABPM. Presence of left ventricular hypertrophy (LVH) was included when it was diagnosed by the reading cardiologist based upon multiple factors including left ventricular mass indexed to height squared and left ventricular wall measurement Z-scores.

### Participants

The study included all patients referred to the Nationwide Children’s Hospital (Columbus, OH) outpatient pediatric nephrology and hypertension clinic to evaluate for possible HTN during the study period. All patients and their caregivers were invited to complete psychosocial screening surveys. Patients were excluded if they had their ABPM for no clear concern of HTN (such as routine evaluation of patients with CKD) or their ABPM data was inconclusive (e.g., they took off the ABPM and there were not sufficient readings).

### Measures

At time of ABPM placement, patients and their caregivers were asked to complete the following rating scales; 1) the Vanderbilt ADHD Diagnostic Parent Rating Scale (21), which is a widely used rating scale to assess clinically significant symptoms of ADHD and commonly co-occurring symptom (i.e., oppositional defiant disorder [ODD], anxiety, and depression). For the purposes of this study, we excluded items assessing conduct disorder as these are infrequent concerns; thus, the rating scale was 31 items. Items are rated from 0 (never) to 3 (very often). The mean response value for each respective subscale was considered. Additionally, patients were considered to have clinically significant symptoms (yes or no; categorically) if parents rated that six or more items in a respective subscale was rated at “often” or “very often,” consistent with the diagnostic use of the Vanderbilt scales. In the current study, the subscales demonstrated good internal consistency (inattention: α = .948; hyperactivity/impulsivity: α = .903; ODD: α = .922; anxiety/depression: α = .921).

2) The Learning, Executive, and Attention Functioning (LEAF) Scale (22), a 55-item freely available (with permission) parent-report rating scale assessing broad executive functioning abilities and related learning skills. Items are rated from 0 (never) to 3 (very often) and then summed in their respective subscale. Per the measure design, scores of 10 or greater in respective subscales are considered the “problem range,” and were thus considered as “clinically significant” in the current study. The LEAF has good reliability and validity evidence, and in our sample all 11 subscales had good to excellent internal consistency: α >.85.

3) The Sleep Disturbances Scale for Children (SDSC) (23), a 26-item scale developed to assess common sleep difficulties (i.e., sleep breathing disorders) as well as provide an overall measure of sleep disturbance that is suitable for clinical screening. Items are scored from 0 (never) to 5 (always), though two items are scored with different anchor descriptions. Scores were considered clinically significant if they corresponded with a T-score greater than 70, as advised by the original scale development. The internal consistency of this scale indicated that all subscales should be interpreted with caution (alphas ranged from.423 to.829), but that the total score demonstrated good internal consistency (α = .877); as such only the total score was used in this study (all subscales are reported in **Supplementary Data**). For the total score, all values of 52 or higher corresponded with a T-score above 70 and thus were considered clinically significant.

Children completed two screening surveys,1) the Center for Epidemiological Studies Depression Scale for Children (CES-DC) (24), a widely used 20-item [rated from 0 (never) to 3 (a lot)] screening survey for childhood and adolescent depression, and 2) the Screen for Child Anxiety Related Disorders (SCARED) (25), a 41-item (rated from 0 [not true] to 3 [very true]) widely used screening survey of symptoms of various common anxiety disorders across childhood and adolescence. These measures were selected as they all can be scored continuously as well as provide clinical cutoffs to indicate level of concern. For the CES-DC, scores of 15 or higher are considered a positive screen for clinical significance; whereas, on the SCARED each subscale has a different value considered clinically significant, with a total score of 25 or greater considered a positive screen for clinical significance. Both measures demonstrated adequate internal consistency; CES-DC: α = .910; subscales of SCARED α >.900, with exception of separation anxiety: α = .790, and school avoidance: α = .734).

### Statistical analysis

We used IBM SPSS Statistics package version 28 to perform descriptive and inferential statistics. To evaluate differences between patients identified with WCH versus HTN, we performed T-tests (for continuous variables) and Chi-Square tests for categorical variables (e.g., clinical elevations versus not clinical elevation) and p-values are provided in the tables. Given the skewness of data, we offer median and interquartile range information on all variables in the **Supplementary Data**. We also conducted Mann Whitney U test for non-parametric data to assess if any study findings would differ, and none did so t-tests were retained as the primary test statistic for the inferential analyses. We did not make any imputations for missing data. Because the available data for each respective variable differed, we indicated relevant sample size for each analysis within the table. A *p* value < 0.05 was considered significant.

## Results

### Medical variables

In total, 591 patients completed the ABPM study, of which we excluded 17 patients (3.0%) who had no hypertension concerns and received the ABPM due to standard monitoring of their CKD or cardiological condition. Additionally, some patients (*n*=22, 3.7%) were excluded due to returning their ABPM without sufficient information (below 40 BP readings), likely due to removing the ABPM soon after placement. Of the remaining 552 patients, 311 patients (56.3%) had ambulatory HTN and 241 (43.6%) had WCH.

The average age at time of enrollment was 14.36 years (SD=3.57 years; range: 4 to 27 years old; 28 patients seen were older than 18 years old) and most of the patients were males (58.8%). There was no significant difference between the group of patients with WCH versus HTN regarding their gender, self- reported race, BMI or metrics of self- reported social determinants of health; however, there was a significant difference in age, wherein those with HTN tended to be slightly older than those with WCH (**Table 1**).

**Table 1:** Demographics of pediatric patients referred to an outpatient nephrology clinic for evaluation of hypertension concerns from 6/1/2020 to 8/31/2021.

Demographic Variables	Entire sample	WCH	HTN	p value
Age years; *M, (SD*) (*n*=547)	14.36 (3.57)	13.97(3.60)	14.67(3.53)	*p*=.025*
Gender (% F) (*n*=548)	41.2	41.9	40.7	*p*=.778
Race (%) (*n*=548)				*p*=.280
White	65.1	62.2	67.4	
Black	20.4	19.5	21.1	
Asian	4.0	5.4	2.9	
American Indian/Alaskan	0.2	0.4	0	
Pacific Islander	0.2	0	0.3	
Multiple Races	6.6	7.9	5.5	
Unknown/Not Reported	3.5	4.6	2.6	
Ethnicity (%)(*n*=548)				*p*=.061
Hispanic	6.0	8.7	3.9	
Not Hispanic	93.2	90.5	95.4	
Other/Unknown	0.7	0.8	0.7	
Primary Language (*n*=548)				*p*=.452
English	91.6	89.6	93.2	
Nepali	1.5	1.7	1.3	
Spanish	4.4	6.2	2.9	
Somali	0.9	0.8	1.0	
Other	1.6	1.7	1.6	
Social Determinants (% concern) (*n*=429)				
Housing Insecurity	0.7	0.5	0.8	*p*=.723
Food Insecurity	1.9	1.6	2.1	*p*=.726
Transportation Insecurity	2.3	1.6	2.9	*p*=.389
Utilities Insecurity	1.6	1.1	2.1	*p*=.420

* Indicates we considered this p-value indicative of a statistically significant difference. P-values were determined based on Independent Samples T-tests for continuous data (age only) and Chi Square tests for all other data. Since all Omnibus Chi-Square tests were not-significant, differences between categories were not explored further. WCH, White coat hypertension; HTN, Hypertension.

Regarding their ABPM data, the mean percentage of nocturnal dipping was 10.49 (*range*: -17.30 to 29.50; *SD*=7.05), the mean percentage of systolic load readings was 22.59 (*range*: 0 to 100; *SD*=23.10) and the mean percentage of diastolic load was 20.76 (*range*: 0 to 100; *SD*=20.26). There were no significant differences between the percentage of nocturnal dipping between patients with WCH and HTN, and as expected the systolic load, diastolic load, and MAP were significantly higher for the HTN group than the WCH group (see **Table 2**).

**Table 2:** Average ambulatory blood pressure monitor data collected (6/1/2020 to 8/31/2021) for pediatric patients evaluated for hypertension in a nephrology outpatient clinic, across diagnoses of WCH and HTN.

ABPM values	Entire sample (*n*=545)	WCH (*n*=239)	HTN (*n*=306)	
	Value, *M* (*SD*)	Value, *M* (*SD*)	Value, *M* (*SD*)	p value
% Nocturnal Dipping	10.49 (7.05)	10.85 (6.144)	10.21 (7.67)	.287
Systolic Load (95%)	22.59 (23.10)	11.58 (11.27)	31.19 (26.14)	<.001*
Diastolic Load (95%)	20.76 (20.26)	12.19 (10.86)	27.45 (23.19)	<.001*
MAP	85.31 (8.64)	82.43 (5.81)	87.57 (9.75)	<.001*

* Indicates we considered this p-value indicative of a statistically significant difference. P-values were determined based on Independent Samples T-Tests. Systolic and or diastolic load is the percentage of systolic/diastolic blood pressure readings that exceed the hypertension threshold (> 95% for children < 13 years of age; >130/80 mmHg daytime, >110/65mmHg nighttime for patients > 13). WCH, white coat hypertension; HTN, hypertension; MAP, mean arterial pressure.

Of the available blood work (**Table 3**), average kidney function was normal in both groups (despite having a statistical significance between the two groups). Prevalence of elevated HbA1c and elevated LDL was high in our cohort (42.7 and 30.2% respectively), with no significant difference between the patients with HTN versus WCH. Additionally, a high proportion of our cohort had elevated BMI (*M* BMI =27.80; *SD* =8.19), with over half (52.2%) having BMI greater than or equal to 95^th^ percentile for age (*Mdn*=95.63; IQR: 73.93 – 98.96); with no significant differences between HTN and WCH groups. When reviewing cardiologist diagnoses, prevalence of LVH was seen in 14.4% of patients with HTN and 17.9% of patients with WCH.

**Table 3:** Lab results for pediatric patients evaluated for hypertension in a nephrology outpatient clinic, across diagnoses of WCH and HTN.

Lab Values	Entire sample			WCH			HTN			p value
	Value, *M* (*SD*)	% Flagged		Value, *M* (*SD*	% Flagged		Value, *M* (*SD*	% Flagged		
		High	Low		Low	High		Low	High	
BMI	81.80 (26.41) (*n*=490)	1.6	52.2	83.29 (25.08) (*n*=211)	1.4	55.9	80.67 (27.37) (*n*=279)	1.8	49.5	*p = .*277
Alb	4.45 (0.45) (*n*=256)	2.5	0.8	4.49 (0.42) (*n*=83)	2.5	1.2	4.43 (0.46) (*n*=173)	2.5	0.6	*p* =.338
BUN	14.4 (7.57) (*n*=286)	0.4	14.6	12.47 (4.17) (*n*=94)	1.1	7.6	15.35 (8.62) (*n*=192)	0	18.3	*p* <.001*
A1C	6.41 (2.37) (*n*=106)	--	42.7	6.12 (1.76) (*n*=45)	--	45.4	6.62 (2.73) (*n*=61)	--	40.7	*p* =.247
LDL	107.92 (36.90) (*n*=100)	11.5	30.2	113.33 (34.16) (*n*=39)	7.7	38.5	104.46 (38.43) (*n*=61)	14.0	24.6	*p* =.243
TG	157.98 (114.47) (*n*=116)	7.0	36.0	135.08 (79.40) (*n*=40)	7.7	28.2	170.04 (128.01) (*n*=76)	6.7	40.0	*p* =.118
eGFR	102.54 (33.57) (*n*=277)	32.1	27.1	108.04 (28.53) (*n*=90)	25.6	30.0	99.89 (35.51) (*n*=187)	35.3	25.7	*p* =.041*
LVH	-- (*n*=164)	--	15.2	-- (*n*=39)	--	17.9	-- (*n*=125)	--	14.4	*p* =.590

* Indicates we considered this p-value indicative of a statistically significant difference. P-values were determined based on Chi Square tests for categorical data (i.e., only LVH, which was categorized as elevated or not elevated) and all other data were compared using the continuous value with Independent Samples T-tests. Data were largely skewed, so Mann Whitney U tests were also performed on all values indicating skewness. BMI refers to BMI percentile for age. WCH, white coat hypertension; HTN, hypertension; BMI, body mass index; Alb, albumin; BUN, blood urea nitrogen; A1C, hemoglobin A1C; LDL, low density lipoprotein cholesterol; TG, triglycerides; eGFR, estimated glomerular filtration rate; LVH, left ventricular hypertrophy.

### Psychosocial screening variables

Regarding the self- administered screening surveys, 472 patients completed the surveys (at least partially: 448 with both parent and patient information, 23 with just parent information, and 1 with just pediatric patient information). Of those who completed the surveys, they were diagnosed with HTN (*n* =265; 56.1%) or WCH (*n* =207; 43.8%) based on their completed ABPM.

Collectively, when these common clinical considerations were combined to consider patients with at least one clinical elevation (i.e., on parent reported ADHD or patient reported total anxiety or depression), just over half of patients screened positive for having at least one clinically significant mental health concern, with virtually identical rates between patients with WCH and HTN (**Table 4**). Looking individually at the psychosocial rating scales, similar lack of differences between groups emerged. Parent-reported symptoms of child ADHD, ODD, or anxiety, and depression symptoms did not differ (**Table 5**), nor did child-reported symptoms of anxiety (**Table 6**) or depression (**Table 7**). Again, on parent report ratings of more functional concerns, such as sleep and executive functioning, no differences were observed across HTN and WCH populations (**Tables 8**, **9**, respectively). Taken together, 306 patients (65.0% of the patients who completed psychosocial screening surveys) were flagged and recommended for behavioral health follow-up.

**Table 4:** Combined data demonstrating patients with a positive screen for a mental health concerns across pediatric patients referred for a hypertension evaluation at a nephrology outpatient clinic.

	Entire sample (*n*=427)	WCH (*n*=190)	HTN (*n*=237)	*X^2^* p-value
Clinical Elevation	% Clinically Elevated	% Clinically Elevated	% Clinically Elevated	
Total Score	50.1% (*n*=214)	50.0% (*n*=95)	50.2% (*n*=119)	*p* =.965

Clinical elevation was calculated by any patient who was deemed clinically elevated on parent ratings on the Vanderbilt of ADHD-IA or ADHD-HI or patient ratings on the SCARED for total anxiety or on the CES-DC for depression (i.e., only four clinical elevation scores were considered). Only participants with complete data on these measures were included in this descriptive analysis. WCH, white coat hypertension; HTN, hypertension.

**Table 5:** Parent report of ADHD, behavior, and mood concerns for pediatric patients evaluated for hypertension concerns at a nephrology outpatient clinic.

	Entire sample (*n*=436)		WCH (*n*=192)		HTN (*n*=244)		t-test p-value
Vanderbilt, parent report	*M* (*SD*)	% Clinically elevated	*M* (*SD*)	% Clinically elevated	*M* (*SD*)	% Clinically elevated	
ADHD-Inattention	0.85 (0.79)	17.2	0.82 (0.79)	16.7	0.87 (0.80)	17.6	*p* =.547
ADHD-Hyperactivity/Impulsivity	0.50 (0.66)	7.1	0.52 (0.70)	8.3	0.48 (0.63)	6.1	*p* =.574
ODD Symptoms	0.63 (0.70)	13.1	0.64 (0.73)	13.5	0.61 (0.68)	12.7	*p* =.658
Anxiety/Depression Symptoms	0.64 (0.70)	16.3	0.62 (0.67)	15.1	0.65 (0.73)	17.2	*p* =.587

The scores are the mean response across items in the respective subscales. P-values were determined based on Independent Samples T-tests for the continuous data (i.e., we used mean value comparisons instead of categorical clinical elevations). WCH, white coat hypertension; HTN, hypertension; Vanderbilt, Vanderbilt ADHD Diagnostic Parent Rating Scale; ADHD, attention-deficit/hyperactivity disorder; ODD, oppositional defiant disorder.

**Table 6:** Self-reported anxiety for pediatric patients evaluated for hypertension concerns at a nephrology outpatient clinic.

	Entire sample (*n*=443)		WCH (*n*=200)		HTN (*n*=243)		*t*-test p-value
SCARED, patient report	*M* (*SD*)	% Clinically elevated	*M* (*SD*)	% Clinically elevated	*M* (*SD*)	% Clinically elevated	
Panic/Somatic	4.06 (4.79)	22.1	4.22 (4.81)	20.5	3.92 (4.78)	23.5	*p*=.508
Generalized anxiety	5.41 (4.72)	27.1	5.15 (4.54)	25.0	5.63 (4.86)	28.8	*p*=.278
Separation anxiety	3.34 (3.32)	30.7	3.40 (3.44)	29.0	3.30 (3.23)	32.1	*p*=.744
Social anxiety	5.61 (4.24)	30.5	5.60 (4.13)	30.5	5.61 (4.34)	30.5	*p*=.974
School avoidance	1.49 (1.79)	21.5	1.48 (1.81)	23.1	1.50 (1.76)	20.2	*p*=.885
Total anxiety	19.91 (15.34)	31.2	19.84 (15.12)	31.0	19.97 (15.55)	31.3	*p*=.931

The mean scores are the sum response across items in the respective subscales. The Panic/Somatic subscale is 13-items (considered elevated at a score of 7 or greater); The Generalized anxiety subscale included 9-items (considered elevated at a score of 9 or greater); The Separation anxiety subscale is 8-items (considered elevated at a score of 5 or greater); The Social Anxiety subscale is 7-items (considered elevated at a score of 8 or greater); The school avoidance subscale is 4-items (considered elevated at a score of 3 or greater); The Total anxiety subscale includes all 41-items (considered elevated at a score of 25 or greater). P-values were determined based on Independent Samples T-tests for the continuous data (i.e., we used mean value comparisons instead of categorical clinical elevations). WCH, white coat hypertension; HTN, hypertension; SCARED, Screen for Childhood Anxiety and Related Disorders.

**Table 7:** Self-reported depression for pediatric patients evaluated for hypertension concerns at a nephrology outpatient clinic.

	Entire sample (*n* = 443)		WCH (*n* = 200)		HTN (*n* = 243)		t-test p-value
CES-DC, patient report	*M* (*SD*)	% Clinically elevated	*M* (*SD*)	% Clinically elevated	*M* (*SD*)	% Clinically elevated	
Total Score	11.42 (9.88)	28.9	11.56 (10.03)	27.5	11.30 (9.77)	30.0	.395

The scores are the sum across subscales. P-values were determined based on Independent SampleT-tests for the continuous data (i.e., we used mean value comparisons instead of categorical clinical elevations). WCH, white coat hypertension; HTN, hypertension; CES-DC, Center for Epidemiological Studies Depression Scale for Children.

**Table 8:** Parent-reported sleep concerns for pediatric patients evaluated for hypertension concerns at a nephrology outpatient clinic.

	Entire sample (*n*=442)		WCH (*n*=200)		HTN (*n*=242)		t-test p-value
Sleep parent report	*M* (*SD*)	% Clinically elevated	*M* (*SD*)	% Clinically elevated	*M* (*SD*)	% Clinically elevated	
Total Score	38.55 (11.46)	12.4	38.72 (10.55)	14.0	38.40 (12.18)	11.2	*p*=.771

The scores are the sum across subscale items. P-values were determined based on Independent Samples T-tests for the continuous data (i.e., we used mean value comparisons instead of categorical clinical elevations). Sleep was based on total score from the *Sleep Disturbances Scale for Children.* WCH, white coat hypertension; HTN, hypertension.

**Table 9:** Parent-reported executive functioning and academic concerns for pediatric patients evaluated for hypertension concerns at a nephrology outpatient clinic.

	Entire sample (*n*=434)		WCH (*n*=196)		HTN (*n*=238)		t-test p-value
LEAF/executive functioning, parent report	*M* (*SD*)	% Clinically elevated	*M* (*SD*)	% Clinically elevated	*M* (*SD*)	% Clinically elevated	
Comprehension and Conceptual Learning	2.58 (3.54)	7.6%	2.62 (3.52)	7.1	2.55 (3.56)	8.0	*p*=.845
Factual Memory	2.69 (3.68)	6.7%	2.79 (3.70)	7.7	2.62 (3.66)	5.9	*p*=.636
Attention	3.87 (4.52)	16.1%	3.86 (4.59)	16.3	3.88 (4.47)	16.0	*p*=.962
Processing Speed	3.74 (4.32)	13.1%	3.58 (4.32)	12.2	3.88 (4.32)	13.9	*p*=.464
Visual-Spatial Organization	3.38 (3.76)	10.4%	3.28 (3.61)	8.7	3.45 (3.88)	11.8	*p*=.633
Sustain Sequential Processing	3.74 (4.23)	13.0%	3.69 (4.25)	13.3	3.78 (4.23)	12.8	*p*=.823
Working Memory	3.93 (4.44)	15.4%	3.77 (4.32)	14.4	4.06 (4.54)	16.2	*p*=.506
Novel Problem-Solving	3.17 (3.76)	7.6%	3.04 (3.71)	8.0	3.28 (3.81)	7.2	*p*=.525
Mathematics Skills	3.58 (4.46)	14.0%	3.51 (4.26)	12.3	3.64 (4.62)	15.3	*p*=.766
Basic Reading Skills	3.09 (4.42)	11.8%	3.05 (4.34)	10.2	3.12 (4.49)	13.2	*p*=.872
Written Expression Skills	3.51 (4.56)	14.0%	3.46 (4.40)	14.4	3.55 (4.70)	13.6	*p*=.835

The scores are the sum across subscale items. P-values were determined based on Independent Samples T-tests for the continuous data (i.e., we used mean value comparisons instead of categorical clinical elevations). WCH, white coat hypertension; HTN, hypertension; LEAF, The Learning, Executive, and Attention Functioning Scale.

## Discussion

WCH is common in children and adolescents during routine clinic visits and recent studies showed that WCH may not be a benign phenomenon, increasing risks of cardiovascular diseases and/or HTN with time (11, 15–18). Our study showed that WCH is common in pediatric patients with concerns of elevated BP in pediatricians’ clinics (found in 44% of patients referred for evaluation of HTN). An unexpected finding was that there were no significant differences in obesity, dyslipidemia, or psychological and neurocognitive screenings between patients with HTN versus WCH.

Across our cohort, a large percentage of patients evaluated for hypertension had elevations in areas of common psychological concerns, regardless of WCH or HTN diagnosis. Our findings suggest that patients with WCH do not have increased behavioral health symptoms compared to patients with HTN. Although more than half of the patients and their parents screened positive for clinically significant psychological concerns, only one fifth of these patients reported currently receiving behavioral health services (data not shown). The presence of psychological concerns such as inattention, anxiety, and depression can have several important implications for both clinical practice and future research. While studies showed the average prevalence of anxiety among children aged 6–18 years is estimated around 15-20% (26), this is seen in 28-30% of our cohort. Early identification and intervention of such concerns can be impactful as behavioral therapy, and possibly medical management, may help address symptoms before they cause significant impairments in daily life. Thus, our findings suggest that due to the high incidence of these concerns, behavioral health support (either embedded or referral based) may be beneficial as a standard part of management of patients with hypertension concerns in pediatric nephrology clinics.

In terms of metabolic risk factors (such as elevated BMI, elevated HbA1c and/or dyslipidemia), our study did not find significant differences between patients with HTN and those with WCH. Specifically, while obesity is a known risk factor for HTN, there was no significant difference in BMI between patients with WCH versus HTN in our cohort. This emphasizes the importance of using an ABPM to confirm the diagnosis of HTN in patients with obesity before initiating further expensive evaluation and management. Interestingly, while LVH was seen in 14.4% of patients with HTN, it was also detected in 17.9% of patients with WCH. It is of note that since significant differences in BMI were not observed between the two groups, it is possible that LVH is related to obesity rather than blood pressure in our sample (27, 28). Our findings support the notion that obesity-related interventions should not be deprioritized in patients with WCH, and perhaps these patients should be considered for pharmacological interventions (14). As other findings indicate, WCH may not be a benign condition and should be evaluated and monitored with time (11, 14, 17, 29). Mancia et al. showed that at 10 years’ follow-up, 42.6% (*P*<.0001) of patients with WCH had developed HTN (17). Another study by Sung et al. showed a hazard ratio of 2.94 for 15-year cardiovascular mortality in patients with WCH compared with patients with prehypertension, even after adjusting for age, sex, BMI and smoking (29). Finally, even when looking at pediatric patients with WCH, Miyashita et al. showed that they are more likely to eventually develop systemic HTN (11), suggesting that those patients need to have a prolonged follow up similar to patient with an established HTN diagnosis. Since there were no significant differences between patients with HTN versus WCH on metabolic considerations, it may be important not to discard WCH as a manifestation of behavioral health issues but rather develop a tailored approach for managing and following patients with WCH.

## Future directions and limitations

Future research should explore if having psychological therapy and social support (or even psychological medications) will help improve the control of HTN and/or WCH in a multicenter prospective study, rather than escalating medical management with BP medications, as some studies find a remission of behavioral concerns with HTN treatment (30). Additional research should continue to explore how psychological functioning and blood pressure are associated temporally, that is, are blood pressure elevations causing concerns for psychosocial functioning, or is it the reverse, that psychological functioning may be an early indicator of blood pressure concerns (31, 32). Future research should also consider if psychosocial factors may help identify patients with WCH that may progress to HTN faster, or if they are correlated with more severe medical outcomes. Finally, future research and clinical considerations should explore the merits of managing patients with WCH, particularly those with comorbid obesity, managing their elevated BMI, LDL, HbA1c to improve the population’s health status.

Our study was limited by the study design, inclusive of being cross-sectional analysis at a single center. Additionally, our study included individuals over the age of 18 years old (i.e., 28 patients were older than 18). Including these patients meant that we had to adapt screening tools and measurements (e.g., BMI percentile) outside the recommended range of interpretation. We decided to err on the side of being inclusive and representative of our patient population, but this may decrease generalizability of our findings. We also used the CKiD formula for all patients (to be consistent with the method of estimating kidney function) as opposed to using the bedside Schwartz formula for patients with no CKD. The psychosocial screening surveys used are also brief screening surveys and not a full diagnostic assessment and some were not fully completed. Replication of this analysis across multiple centers with more diverse populations would help determine whether the lack of observed differences in metabolic and psychosocial characteristics between HTN and WCH pediatric patients is consistent across settings.

In summary, our study demonstrated little difference between pediatric patients with HTN versus WCH in terms of psychological or metabolic factors. Our study highlighting the importance of recognizing the high prevalence of psychological concerns, suggesting the importance of screening our patients and when needed, having dedicated mental and behavioral health support, to provide our patients with the best medical management and achieve well-rounded patient care.

## Data Availability

The datasets presented in this article are not readily available. Requests to access the datasets should be directed to mahmoud.kallash@nationwidechildrens.org.
