# Supplementary material for: Prevalence of psychological and metabolic concerns in children with white coat and ambulatory hypertension
Source: Front Nephrol. 2026 Jun 8;6:1699959. doi: 10.3389/fneph.2026.1699959 (PMC13284145; doi:10.3389/fneph.2026.1699959)
Supplement: Supplementary file 1 [file Table1.docx]

**Supplemental Data :**

**Supplemental Table 1.** Subscales of the Parent-Report of Sleep Concerns (with poor internal reliability) for pediatric patients evaluated for hypertension concerns at a nephrology outpatient clinic

|  | Entire Sample  (*n*= 442) | | WCH  (*n*= 200) | | HTN  (*n*= 242) | | t-test  p-value |
| --- | --- | --- | --- | --- | --- | --- | --- |
| **Sleep, Parent Report** | *M* (*SD*) | % Clinically Elevated | *M* (*SD*) | % Clinically Elevated | *M* (*SD*) | % Clinically Elevated |  |
| Dx of Initiating/Maintaining Sleep | 12.92(5.08) | 19.5 | 13.10(4.88) | 20.5 | 12.78(5.25) | 18.6 | *p* = .518 |
| Sleep Breathing Dx | 4.12(1.82) | 10.8 | 4.03(1.77) | 9.5 | 4.21(1.87) | 11.8 | *p* = .303 |
| Dx of Arousal | 3.47(1.09) | 6.6 | 3.58(1.15) | 8.5 | 3.38(1.03) | 5.0 | *p* = .065 |
| Sleep-Wake Transition Dx | 8.34(3.26) | 8.8 | 8.24(2.89) | 8.5 | 8.43(3.54) | 9.1 | *p* = .529 |
| Dx of Excessive Somnolence | 7.10(2.90) | 7.3 | 7.06(2.74) | 7.0 | 7.13(3.03) | 7.5 | *p* = .793 |
| Sleep Hyperhidrosis | 2.70(1.60) | 4.3 | 2.76(1.67) | 4.5 | 2.65(1.53) | 4.2 | *p* = .452 |
| Total Score | 38.55(11.46) | 12.4 | 38.72(10.55) | 14.0 | 38.40(12.18) | 11.2 | *p* = .771 |
| Notes. The scores are the sum across subscale items. P-values were determined based on Independent Samples T-tests for the continuous data (i.e., we used mean value comparisons instead of categorical clinical elevations). WCH = white coat hypertension; HTN = hypertension | | | | | | | |

**Supplemental Table 2. Medians across all variables assessed in the full report to provide context and comparison for skewed variables across pediatric patients evaluated for hypertension concerns at a nephrology outpatient clinic**

|  | Entire Sample | WCH | HTN |
| --- | --- | --- | --- |
| Domains across Instruments | *Mdn* (*IQR*) | *Mdn* (*IQR*) | *Mdn* (*IQR*) |
| **ABPM** |  |  |  |
| % Nocturnal Dipping | 11.10 (6.50-15.28) | 11.00 (6.70-15.40) | 10.21 (6.20-15.20) |
| Systolic Load (95%) | 15.00 (5.30-32.45) | 8.30 (2.90-18.40) | 23.95 (9.40-50.00) |
| Diastolic Load (95%) | 14.30 (5.70-30.95) | 8.80 (5.10-17.60) | 22.05 (8.30-40.60) |
| MAP | 85.00 (80.25-90.00) | 83.00 (79.00-86.00) | 88.00 (82.00-93.00) |
| **Labs** |  |  |  |
| BMI | 95.63 (73.93-98.96) | 95.73 (77.30-98.95) | 94.79 (73.27-98.97) |
| Alb | 4.50 (4.20-4.70) | 4.50 (4.20-4.80) | 4.50 (4.20-4.70) |
| BUN | 12.50 (10.00-16.00) | 12.00 (10.00-12.45) | 13.00 (10.00-17.00) |
| A1C | 5.50 (5.20-6.40) | 5.50 (5.15-6.30) | 5.50 (5.20-6.45) |
| LDL | 103.00 (88.25-123.00) | 108.00 (97.00-128.00) | 99.00 (78.50-119.00) |
| TG | 122.50 (84.00-194.75) | 102.00 (74.00-184.50) | 135.00 (89.50-218.75) |
| eGFR | 103.55 (83.77-122.66) | 103.71 (89.21-124.70) | 103.47 (78.65-121.70) |
| **VANDERBILT** |  |  |  |
| ADHD-Inattention | 0.65 (0.22-1.33) | 0.59 (0.11-1.44) | 0.67 (0.22-1.33) |
| ADHD-Hyperactivity/Impulsivity | 0.22 (0.00-0.67) | 0.22 (0.00-0.78) | 0.22 (0.00-0.67) |
| ODD Symptoms | 0.38 (0.00-1.00) | 0.38 (0.00-1.00) | 0.38 (0.03-0.88) |
| Anxiety/Depression Symptoms | 0.43 (0.14-1.0) | 0.43 (0.14-1.0) | 0.43 (0.14-1.00) |
| **SCARED** |  |  |  |
| Panic/Somatic | 2.00 (1.00-6.00) | 2.00 (1.00-6.00) | 2.00 (0.00-6.00) |
| Generalized anxiety | 4.00 (1.00-9.00) | 4.00 (1.00-8.75) | 4.00 (1.00-9.00) |
| Separation anxiety | 2.00 (1.00-5.00) | 2.00 (1.00-5.00) | 2.00 (1.00-5.00) |
| Social anxiety | 5.00 (2.00-9.00) | 5.00 (2.00-9.00) | 5.00 (2.00-9.00) |
| School avoidance | 1.00 (0.00-2.00) | 1.00 (0.00-2.00) | 1.00 (0.00-2.00) |
| Total anxiety | 16.00 (8.00-29.00) | 16.00 (8.00-29.00) | 18.00 (8.00-29.00) |
| CES-DC |  |  |  |
| Total Score | 9.00 (4.00-16.00) | 9.00 (4.00-16.00) | 9.00 (4.00-16.00) |
| **LEAF** |  |  |  |
| Comprehension and Conceptual Learning | 1.00 (0.00-4.00) | 1.00 (0.00-4.00) | 1.00 (0.00-4.00) |
| Factual Memory | 1.00 (0.00-5.00) | 1.00 (0.00-5.00) | 1.00 (0.00-5.00) |
| Attention | 2.00 (0.00-7.00) | 2.00 (0.00-5.00) | 2.00 (0.00-7.00) |
| Processing Speed | 2.00 (0.00-6.00) | 2.00 (0.00-5.00) | 2.00 (0.00-7.00) |
| Visual-Spatial Organization | 2.00 (0.00-5.00) | 2.00 (0.00-5.00) | 2.00 (0.00-6.00) |
| Sustain Sequential Processing | 2.00 (0.00-6.00) | 2.00 (0.00-6.00) | 2.00 (0.00-7.00) |
| Working Memory | 2.00 (0.00-7.00) | 2.00 (0.00-6.00) | 2.00 (0.00-7.00) |
| Novel Problem-Solving | 2.00 (0.00-5.00) | 2.00 (0.00-4.75) | 2.00 (0.00-5.00) |
| Mathematics Skills | 2.00 (0.00-5.00) | 2.00 (0.00-5.00) | 2.00 (0.00-6.00) |
| Basic Reading Skills | 1.00 (0.00-5.00) | 1.00 (0.00-5.00) | 1.00 (0.00-5.00) |
| Written Expression Skills | 1.00 (0.00-5.00) | 2.00 (0.00-5.00) | 1.00 (0.00-5.00) |
| **SDSC** |  |  |  |
| Total Sleep | 35.00 (31.00-43.00) | 35.00 (31.00-44.00) | 35.00 (30.00-42.25) |
| WCH = white coat hypertension; HTN = hypertension; MAP = mean arterial pressure; BMI= body mass index; Alb = albumin; BUN = blood urea nitrogen ; A1C = hemoglobin A1C ; LDL = low density lipoprotein cholesterol; TG = triglycerides ; eGFR = estimated glomerular filtration rate ; LVH = left ventricular hypertrophy; Vanderbilt = Vanderbilt ADHD Diagnostic Parent Rating Scale ; ADHD = attention-deficit/hyperactivity disorder; ODD = oppositional defiant disorder; SCARED = Screen for Childhood Anxiety and Related Disorders; CES-DC = Center for Epidemiological Studies Depression Scale for Children; LEAF = The Learning, Executive, and Attention Functioning Scale; SDSC= Sleep Disturbances Scale for Children | | | |
